# Medial patellofemoral ligament fixation with suture tape augmentation decreases lateral patellar motion without changing contact pressure

**DOI:** 10.1002/jeo2.70240

**Published:** 2025-04-16

**Authors:** Nima Rezaie, Wesley R. Stroud, David P. Beason, Jonathan S. Slowik, Travis Dias, Grant M. Uldrich, Glenn S. Fleisig, Jeffrey R. Dugas

**Affiliations:** ^1^ American Sports Medicine Institute Birmingham Alabama USA; ^2^ Andrews Sports Medicine and Orthopaedic Center Birmingham Alabama USA

**Keywords:** biomechanics, MPFL augmentation, Schöttle's point

## Abstract

**Purpose:**

Medial patellofemoral ligament (MPFL) reconstruction has been the standard of care for recurrent patellar dislocations and chronic patellar instability. MPFL repair has been used as an alternative surgical option. The purpose of this study was to assess patellar stability and patellofemoral contact mechanics following MPFL fixation with suture tape augmentation. We hypothesized that lateral patellar motion would be reduced.

**Methods:**

In twelve cadaver knees, a hole was drilled near the midpoint of the medial patella. Three locations were drilled on the femur Schöttle's point, 1 cm anterior to Schöttle's point and 1 cm proximal to Schöttle's point. Each knee was then held at 30° of knee flexion, and the patella was subjected to a physiologic lateral force. The resulting motion was measured, and patellofemoral contact forces were recorded. This process was performed with the MPFL torn and then bolstered with suture tape augmentation anchored centrally in the medial patella and each of the three femur hole locations.

**Results:**

All MPFL augmentations provided significantly less lateral patellar motion compared to the torn condition. Contact area was significantly greater in the augmented condition than in the torn condition, but no statistical differences were observed in patellofemoral contact pressure. No significant differences in lateral patellar motion, contact pressure or contact area were found between femoral anchor positions.

**Conclusions:**

MPFL fixation with suture tape augmentation significantly decreased lateral patellar motion compared to the torn condition without causing significant changes in contact pressures within the patellofemoral joint.

**Level of Evidence:**

N/A.

**Abbreviations**
ANOVA: analysis of variance
cm: centimetre
HSD: honest significant difference
mm: millimetre
MPa: megaPascal
MPFL: medial patellofemoral ligament
N: Newton
PEEK: polyether ether keytone
PMMA: polymethyl methacrylate

## INTRODUCTION

The medial patellofemoral ligament (MPFL) serves as the primary restraint to lateral patellar translation in the first 20–30° of knee flexion [1, 24, 28, 49, 50]. It originates on the posterior aspect of the femoral medial epicondyle and inserts along the superomedial border of the patella [9, 48, 50]. Kinematically, the MPFL balances the lateral retinaculum and aligns the patella within the trochlear groove [1, 4, 10]. Studies have shown that the anatomic MPFL is relatively tight in extension and early flexion, and nearly isometric beyond 30° of knee flexion [26]. In the setting of patellar dislocations or subluxations, the MPFL is commonly torn or attenuated due to the fact that this structure accounts for 50%–60% of the lateral restraining force [15, 37, 39]. In fact, studies have shown that the MPFL is damaged in up to 89%–100% of patellar dislocations, leading to an increased risk for recurrence and persistent lateral patellar instability [14, 36, 42, 46]. MPFL tears account for roughly 3% of all knee injuries and are most prevalent in females between 10 and 17 years of age [27, 41]. While a first‐time patellar dislocation is generally treated nonoperatively, indications for surgical intervention include recurrent patellar instability and failed conservative management.

MPFL reconstruction is the most widely used surgical option for patellar instability and can be performed in isolation, or in combination with other procedures to address concomitant pathology surrounding the patellofemoral joint [1, 49, 51]. Multiple reconstruction techniques using various tendon grafts and fixation methods have been described in the literature with good to excellent outcomes regarding patellar stability and quality of life [7, 25, 30, 44]. Although reconstruction adequately restores the medial soft tissues to provide patellar stability, it is not without complications [24, 48]. Studies have shown that autologous tendon graft harvest around the knee can lead to persistent pain and alterations in gait patterns and knee joint kinematics [23, 57], although the use of allografts could be seen to reduce this comorbidity.

In the acute setting, MPFL repair has been used as an alternative surgical option with less patient donor site morbidity, although inferior clinical outcomes and high failure rates have been shown in the initial literature [8, 11, 15, 16, 33, 35, 40]. In 2019, Hopper et al. introduced MPFL repair with suture tape augmentation. They proposed that the addition of suture tape as a secondary stabilizer allows native ligament healing and early mobilization while avoiding unnecessary morbidity of graft harvest [24]. Recent experimental work on cadaveric knees has shown that MPFL repair augmented with suture tape recreates native conditions with regard to patellar stability [60].

Graft isometry is highly regarded as a consistent and crucial element in a successful MPFL reconstruction. Improper graft positioning, as with anterior cruciate ligament reconstruction, has been noted to result in graft failure, over tensioning, and degenerative disease leading to poor outcomes [1, 6, 17, 47, 55]. Schöttle et al. established a radiographic landmark that is now widely used to reproducibly position an anatomic femoral tunnel for the MPFL [45]; however, much like the anatomy of the MPFL itself, the ideal graft positioning between femoral and patellar tunnels remains a debated topic [21, 26, 35, 51, 54, 58]. Minimal research has been performed to determine the optimal tunnel positions for MPFL repair with suture tape augmentation.

The purpose of this study was to determine viable locations for MPFL repair with regard to isometry and to use these locations to assess patellar stability and patellofemoral contact mechanics with the MPFL torn and following MPFL fixation (without direct repair) with suture tape augmentation. We hypothesized that MPFL augmentation would reduce lateral patellar motion and increase the patellofemoral contact area. Furthermore, we hypothesized that patellar motion and patellofemoral contact area after MPFL augmentation would vary for different femoral anchor locations.

## METHODS

### Specimen preparation

Twelve fresh‐frozen knees were procured (Science Care) with a maximum age of 60 years. Knees from donors with a known medical history of joint abnormalities and/or previous surgery or fracture were excluded from the study prior to being procured. Specimens were stored frozen at −20°C and thawed for 24 h at room temperature prior to testing. One specimen had to be removed from the study because it was not able to achieve a full range of motion (i.e., knee flexion of 120° for the purposes of our study), possibly due to arthritis or other knee pathology. The remaining eleven specimens representing the final data set had an average age of 49 ± 9 years (range 32–60 years) and comprised six males and five females.

For each tested specimen, a longitudinal incision was made along the medial border of the patella, and dissection was carried out to expose the MPFL. A separate incision was made over the medial femoral epicondyle, and dissection was carried down to the MPFL origin. The MPFL was transected directly off the medial border of the patella to maintain consistency between each dissection.

### Patella and femur hole locations

**Figure 1:**
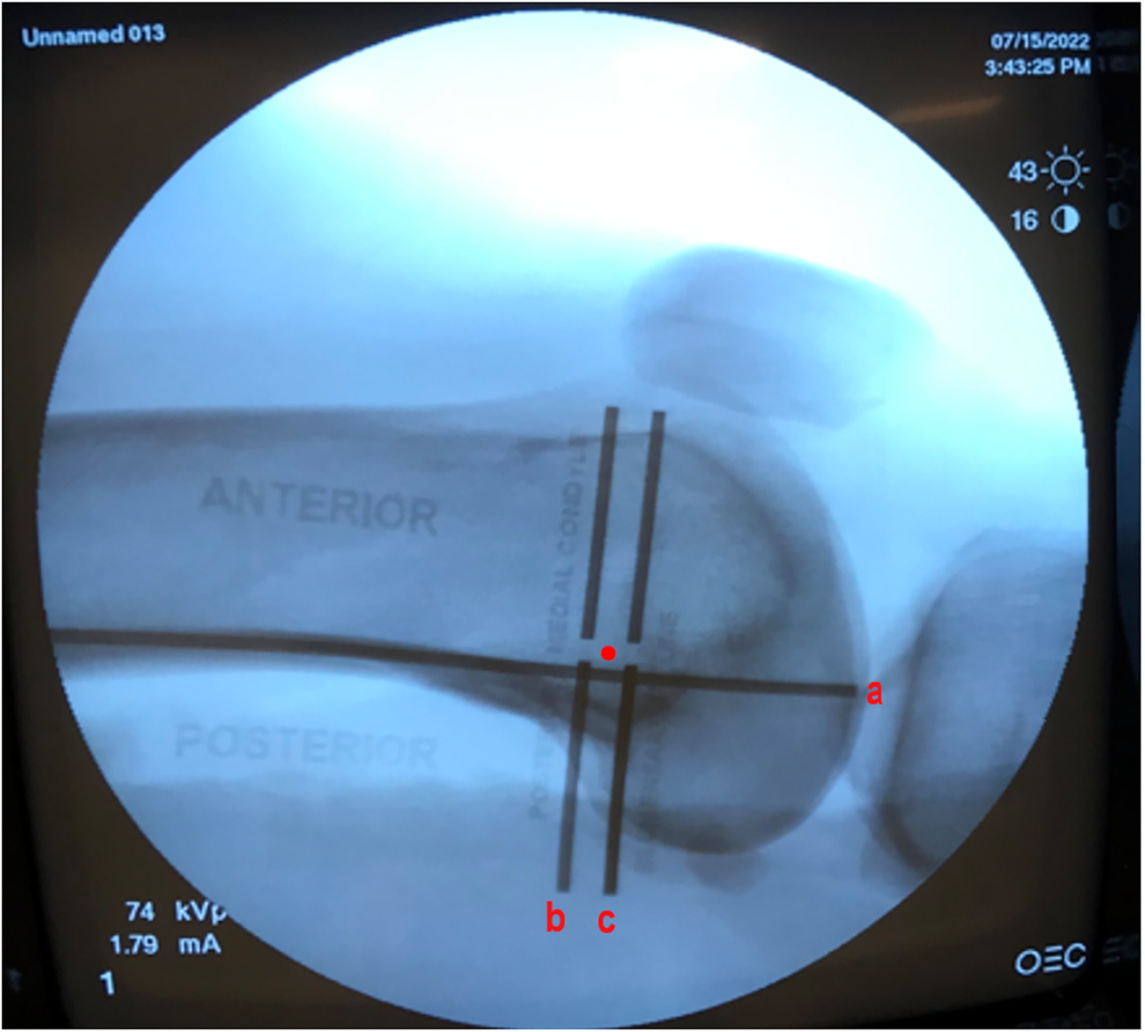
Fluoroscopic image of a left knee with the radiolucent MPFL guide placed in the proper position to identify Schöttle's point. The insertion point (red dot) is approximately (a) 1 mm anterior to the posterior cortex extension line, (b) 2.5 mm distal to the posterior articular border of the medial femoral condyle and (c) proximal to the level of the posterior point of Blumensaat's line [3]. MPFL, medial patellofemoral ligament.

**Figure 2:**
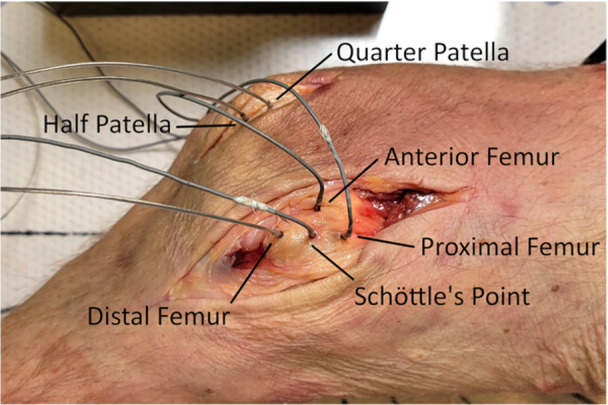
Two patellar and four medial femoral hole locations with electromagnetic microsensors in place in the right knee.

A pilot test was conducted to determine patella and femur hole locations for biomechanical testing. Two holes were drilled on the patella—one at the superior‐inferior midpoint (i.e., ‘half’) and the other at the midpoint between the first hole and the superior pole (i.e., ‘quarter’). Four holes were drilled on the femur—at Schöttle's point as well as 1 cm anterior, 1 cm distal and 1 cm proximal to Schöttle's point. All holes were located and drilled under C‐arm fluoroscopy (Figure 1) and with the aid of an MPFL template (Arthrex). A 1.8‐mm electromagnetic microsensor (Polhemus) was inserted into each femur and patella hole (Figure 2) for pilot testing.

Each knee was then placed lateral side down onto a table with a goniometer‐derived angle map. Static muscle loads were applied to the hamstring (30 N) and all heads of the quadriceps as a single unit (60 N) to ensure patellar stability during testing. Static loads were applied using hanging weights suspended from a stainless steel cable, which was sutured into a central facial portion of each muscle with the aid of nylon fabric, as has been done previously [34]. The femur was then held in a fixed position using a custom fixture, and the knee was manually flexed throughout the entire range of motion (0–120°). Position data were collected at 15° increments, and the Euclidean (i.e., three‐dimensional straight‐line) distances between all eight possible pairings of femoral and patellar hole locations were determined.

For all four femoral locations, knee flexion created more change in distance to the quarter patella than to the half patella. For both patella locations, knee flexion created more change in distance to the distal femur location than the other three femur locations. Therefore, the half patella hole location and three femur locations (anterior femur, proximal femur and Schöttle's point) were included in the biomechanical testing.

### Biomechanical testing

**Figure 3:**
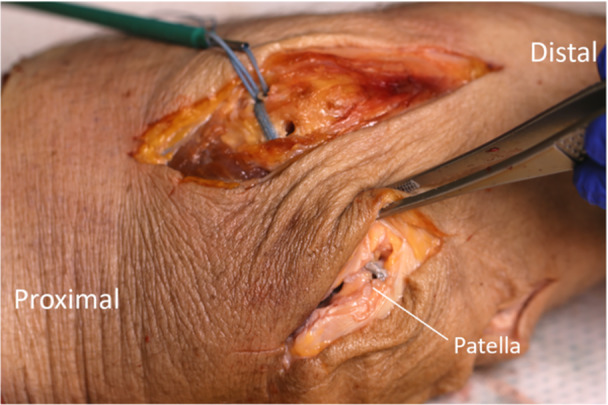
Suture tape in place between the patellar hole and Schöttle's point femoral hole on the right knee.

Using the same knees, the existing medial incision from the superior to inferior aspect of the patella was transected with a medial arthrotomy from the vastus medialis oblique insertion to the patellar tendon origin. A sub‐retinacular tunnel was created between the patellar and femoral incisions to allow for the passage of the suture tape (FiberTape®, Arthrex, Inc.) during the augmentation (Figure 3). An arthrotomy was made through the superior patellar capsule underneath the quadriceps tendons and musculature, and the patellar tendon was longitudinally split to allow for later passage and placement of pressure sensors. The existing holes (where applicable as stated above) were further drilled and tapped for placement of 4.75‐mm suture anchors (4.75‐mm SwiveLock® Arthrex, Inc.) to hold the suture tape on the femur and patella.

**Figure 4:**
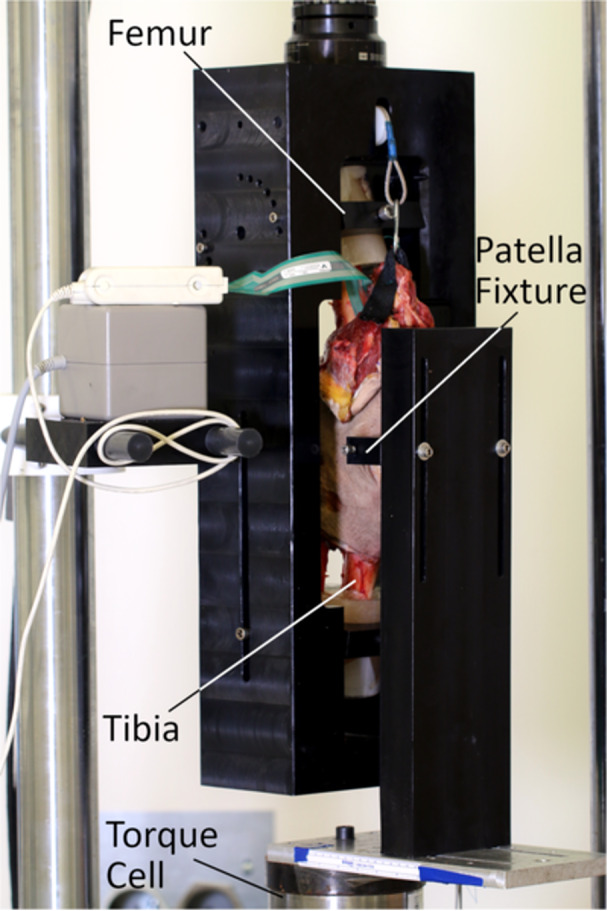
Biomechanical testing setup. Note that the patella is attached to a separate component of the fixture, which is held stationary and fixed to the torque cell, while the rest of the knee is attached to the rotating actuator.

With the MPFL having been cut, the femur and tibia‐fibula of each knee were then potted in plastic cylinders using polymethyl methacrylate (PMMA) and placed into a biomechanical testing apparatus (Figure 4) attached to a servo‐hydraulic test frame actuator (MiniBionix II, MTS Systems). Based upon previous studies assessing the optimal knee position for MPFL repair/reconstruction [9, 10, 26], knee flexion was fixed at 30° in this customized testing apparatus. A Tekscan 4205 pressure sensor (Tekscan) was carefully guided underneath the quadriceps via a hemostat through the longitudinal split in the patellar tendon and placed within the patellofemoral joint. The quadriceps muscle/tendon was then tensioned with a 30‐N static hanging weight via a dual‐pulley system. This testing apparatus allowed the femur and tibia‐fibula to move in concert, while the patella was fixed to a torque cell. The knee was then subjected to 10 cycles of 75‐N lateral force by calculating an input torque equal to 75 N multiplied by the measured distance between the patellar fixation point to the rotation centre of the torque cell. The resulting lateral arc of motion, as well as patellar contact area, pressure, force, peak pressure and peak force, were measured and/or calculated from the 10th cycle.

Following testing in the torn condition, each knee was placed lateral side down onto a table, and a polyether ether ketone (PEEK) 4.75‐mm SwiveLock® suture anchor (Arthrex, Inc.) loaded with the suture tape was placed in the patella, where it would remain for the duration of testing. The suture tape was then brought through the sub‐retinacular tunnel and loaded in another PEEK 4.75‐mm anchor. With the knee held in 30° of flexion using a goniometer, the black laser line on the suture anchor inserter was held over one of the femoral holes allowing a reproducible amount of suture tape and tension to be placed prior to inserting the anchor. The knee was again placed in the biomechanical testing apparatus and tested as previously described, followed by the femoral anchor being removed and seated in a different femoral location. The test process was repeated until all three femoral locations received a suture tape augmentation and biomechanical testing. The test order was evenly distributed between the femoral locations, and each location was tested first, second or third approximately an equal number of times.

### Statistical analyses

Differences between torn and augmented conditions were statistically analyzed using one‐way analysis of variance (ANOVA) with repeated measures, and Tukey's honest significant difference (HSD) for pairwise comparisons. The overall effects of femoral anchor location were statistically analyzed using two‐way (torn‐vs‐augmented, femur anchor location) ANOVA with repeated measures of Tukey's HSD. The level of statistical significance for all analyses was set at *p* ≤ 0.05.

## RESULTS

**Figure 5:**
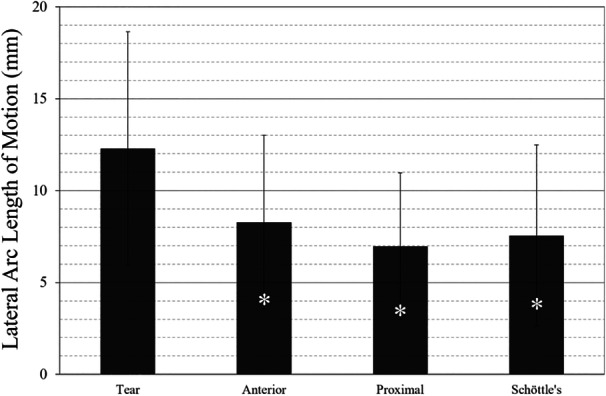
Suture tape augmentation, when spanning the half‐patella and all three femoral anchor locations, resulted in improved (i.e., reduced) lateral motion compared to the torn condition for anterior (*p* = 0.0094), proximal (*p* = 0.0005) and Schöttle's (*p* = 0.0019). Significant differences from torn are marked (*) within the bars.

Overall results of biomechanical testing (Table 1) showed no effect (*p* = 0.70) of femur location on lateral motion, as measured by arc length, but a significant effect (*p* < 0.001) of condition (i.e., torn vs. augmented). One‐way ANOVA showed significantly reduced (*p* < 0.01) lateral motion for all femoral anchor locations, when compared to the torn condition (Figure 5); however, as also indicated by the two‐way ANOVA, augmentations with varied femoral anchor locations were not significantly different (*p* > 0.69) from each other. Contact area was significantly affected (*p* = 0.024) by condition, with higher contact areas in the augmented condition. Contact pressure showed a non‐significant trend (*p* = 0.058), favouring the augmented condition. No other significant differences (*p* > 0.53) were found in contact force, peak pressure or peak force.

**Table 1:** Results from biomechanical testing.

		MPFL augmented from patella to		
	Torn MPFL	Schöttle's point	Anterior femur	Proximal femur
Lateral motion (mm)*	12.3 ± 6.4	7.6 ± 4.9	8.3 ± 4.7	7.0 ± 4.0
Contact pressure (MPa)	0.45 ± 0.16	0.40 ± 0.14	0.38 ± 0.15	0.41 ± 0.11
Contact force (N)	61.1 ± 26.1	61.9 ± 26.1	55.8 ± 30.0	65.2 ± 23.7
Contact area (mm^2^)*	156 ± 67	194 ± 60	171 ± 94	202 ± 85
Peak pressure (MPa)	0.82 ± 0.31	0.70 ± 0.31	0.67 ± 0.29	0.81 ± 0.30
Peak force (N)	12.0 ± 4.5	10.2 ± 4.5	9.7 ± 4.5	11.8 ± 4.3

*Note*: Values represent mean ± standard deviation. There were no statistical differences between the three augmentations with varied femoral anchor points.

Abbreviation: MPFL, medial patellofemoral ligament.

* Significant difference (*p* < 0.05) between torn and augmented MPFL.

## DISCUSSION

As hypothesized, MPFL fixation with suture tape augmentation achieved a significant reduction in the lateral translation of the patella. Comparing our study with the few articles that have investigated MPFL with suture tape augmentation, the present study utilized an adequate number of cadaveric specimens and made use of an accurate, reproducible method for assessing patellofemoral stability and contact mechanics with and without MPFL augmentation. The lack of significant differences observed in patellofemoral contact mechanics between the different femoral anchor points did not indicate biomechanical superiority for any particular pair of hole locations.

As mentioned before, MPFL repair has been shown to have less patient donor site morbidity; however, inferior clinical outcomes and high failure rates have been shown in the initial literature [8, 11, 15, 16, 33, 35, 40]. Because of the concomitant procedures associated with MPFL reconstruction, it is often difficult to compare results among studies. Overall, however, surgical treatment of patellar instability, including MPFL reconstruction rates, has risen over the past two decades [2, 22]. Numerous studies and systematic reviews have shown that MPFL reconstruction has generally favourable clinical outcomes compared to MPFL repair and nonoperative management [18, 31, 32, 43, 44, 50]; however, it is worth noting that a recent review concluded that between MPFL repair and nonoperative treatment, MPFL repair appears to be relatively advantageous [29]. Despite these benefits in reducing instability, MPFL reconstruction has also been associated with higher complication rates [2, 38, 48, 50]. Given this conflict, it is prudent to analyze the factors which may contribute to suboptimal conditions. Many areas have been investigated to elucidate where improvements can be made, including predisposing factors, graft selection, fixation method and tunnel positioning [13, 17, 19, 48, 52]. There remains a lack of consensus on a standard surgical technique largely due to the multifactorial nature of an MPFL injury and the heterogeneous population that undergoes MPFL reconstruction [5, 12, 56].

Recently, there has been a renewed interest in MPFL repair with studies showing acceptable results. The addition of suture tape as a secondary stabilizer has been proposed to allow native ligament healing and early mobilization while avoiding unnecessary morbidity of graft harvest [24] and has also been shown to be an effective treatment option in paediatric patients, especially those with patellofemoral instability [20]. Another advantage is the utilization of smaller implants, which may be less likely to violate the epiphyseal plate of the distal femur in paediatric patients suffering from patellar instability. While this may be a viable alternative for restoring patellar stability, there is concern that this technique may over‐constrain the patellofemoral joint leading to potential pain, loss of motion and patellofemoral cartilage damage.

The current study was not without its limitations. When drilling hole locations, the 1‐cm relative distances between the four femoral holes remained constant, rather than scaled up or down based on actual specimen size. This allowed for the most consistent practical application of these relative distances. Perhaps most notably, patellofemoral stability and contact mechanics were measured only at 30° of knee flexion. This may have limited our ability to detect significant differences between the three femoral anchor points, as studies have shown femoral tunnel malposition on the femur leads to greater change in graft length at varying degrees of knee flexion [49, 51, 53]. Also, as a cadaveric study, our measurements were obtained at the time of fixation, thus conclusions pertaining to long‐term results cannot be drawn due to potential loosening of the suture tape over time secondary to cyclic loading. Due to our dissection to assess isometry, we violated several anatomical structures of the knee, namely the MPFL at its patellar insertion, which may have altered the native knee joint biomechanics. This prevented us from assessing the MPFL in its intact state, which would have provided additional valuable information. Repeat testing of the cadaveric knees may have also compromised the soft‐tissue integrity secondary to changes in temperature as well as placement and removal of the pressure sensor, which may have affected our results. This was partially accounted for by evenly distributing the test order of the different femoral anchor points. The radiographic method for identifying the location of Schöttle's point has been shown to not always correlate with the anatomic attachment of the MPFL [59]. We chose this method as it is reproducible and widely used in clinical practice. Finally, the anatomy of our cadaveric knees must be considered as a possible limitation. These knees presumably had normal anatomy with no patellar maltracking or concomitant pathology, which limits their clinical application in patients with co‐pathologies such as trochlear dysplasia or patellar malalignments.

Despite limitations inherent to our study design, our study provides valuable information on patellofemoral biomechanics following MPFL fixation with suture tape augmentation using different anchor points on the femur. Further studies are necessary to investigate the durability of the stabilizing effect of suture tape augmentation with direct repair of the ligament, its influence on healing of the native MPFL, and long‐term clinical outcomes. Testing at multiple knee flexion angles could also provide additional valuable information.

In conclusion, MPFL fixation with suture tape augmentation significantly decreased lateral patellar motion and increased patellofemoral contact area compared to the torn condition. There was no significant difference in patellofemoral contact pressure between torn and augmented conditions. Furthermore, there were no significant differences between anterior femoral, Schöttle's point or proximal femoral anchor positions in regard to lateral patellar translation or contact mechanics.

## AUTHOR CONTRIBUTIONS

Study conception and design is attributed to Jeffrey R. Dugas, Glenn S. Fleisig, David P. Beason and Nima Rezaie. Material preparation, data collection and analysis were performed by Nima Rezaie, Wesley R. Stroud, David P. Beason, Jonathan S. Slowik, Travis Dias and Grant M. Uldrich. The first draft of the manuscript was written by Nima Rezaie, Wesley R. Stroud, David P. Beason, Jonathan S. Slowik, Travis Dias and Grant Uldrich. All authors commented on previous versions of the manuscript. All authors read and approved the final manuscript.

## CONFLICT OF INTEREST STATEMENT

Dr. Nima Rezaie declares that educational support is available from Arthrex (the study sponsor), Fones Marketing Management and Smith & Nephew. Dr. Wesley R. Stroud declares educational support from Prime Surgical and Smith & Nephew and reimbursement for travel and lodging from Zimmer Biomet. Dr. Jeffrey R. Dugas is/was a paid consultant for Arthrex (the study sponsor), Bioventus, DJO and Royal Biologics. Dr. Jeffrey R. Dugas has received non‐consulting and/or speaking fees, reimbursement for travel and lodging, and royalties/licences for Arthrex (the study sponsor). Dr. Jeffrey R. Dugas declares educational support, hospitality and royalties/licences from DJO. The remaining authors declare no conflicts of interest.

## ETHICS STATEMENT

Ethics approval (i.e., IRB) was not required by our institution due the study did not involve live human subjects.

## Data Availability

The data that support the findings of this study are available from the corresponding author upon reasonable request.

## References

[1] Amis AA , Firer P , Mountney J , Senavongse W , Thomas NP . Anatomy and biomechanics of the medial patellofemoral ligament. Knee. 2003;10(3):215–220. 12893142 10.1016/s0968-0160(03)00006-1

[2] Arshi A , Cohen JR , Wang JC , Hame SL , McAllister DR , Jones KJ . Operative management of patellar instability in the United States: an evaluation of national practice patterns, surgical trends, and complications. Orthop J Sports Med. 2016;4(8):2325967116662873. 27631015 10.1177/2325967116662873 PMC5010099

[3] Arthrex . Medial patellofemoral ligament (MPFL) reconstruction surgical technique. 2021.

[4] Bicos J , Fulkerson JP , Amis A . Current concepts review: the medial patellofemoral ligament. Am J Sports Med. 2007;35(3):484–492. 17303819 10.1177/0363546507299237

[5] Black SR , Meyers KN , Nguyen JT , Green DW , Brady JM , Maher SA , et al. Comparison of ligament isometry and patellofemoral contact pressures for medial patellofemoral ligament reconstruction techniques in skeletally immature patients. Am J Sports Med. 2020;48(14):3557–3565. 33135907 10.1177/0363546520966609

[6] Bollier M , Fulkerson J , Cosgarea A , Tanaka M . Technical failure of medial patellofemoral ligament reconstruction. Arthroscopy. 2011;27(8):1153–1159. 21664791 10.1016/j.arthro.2011.02.014

[7] Bouras T , U E , Brown A , Gallacher P , Barnett A . Isolated medial patellofemoral ligament reconstruction significantly improved quality of life in patients with recurrent patella dislocation. Knee Surg Sports Traumatol Arthrosc. 2019;27(11):3513–3517. 30820603 10.1007/s00167-019-05447-w

[8] Bryant J , Pandya N . Medial patellofemoral ligament repair restores stability in pediatric patients when compared to reconstruction. Knee. 2018;25(4):602–608. 29886008 10.1016/j.knee.2018.05.004

[9] Buckens CFM , Saris DBF . Reconstruction of the medial patellofemoral ligament for treatment of patellofemoral instability: a systematic review. Am J Sports Med. 2010;38(1):181–188. 19966098 10.1177/0363546509353132

[10] Burrus MT , Werner BC , Conte EJ , Diduch DR . Troubleshooting the femoral attachment during medial patellofemoral ligament reconstruction: location, location, location. Orthop J Sports Med. 2015;3(1):1–8. 10.1177/2325967115569198 PMC4555580 26535373

[11] Camp CL , Krych AJ , Dahm DL , Levy BA , Stuart MJ . Medial patellofemoral ligament repair for recurrent patellar dislocation. Am J Sports Med. 2010;38(11):2248–2254. 20716682 10.1177/0363546510376230

[12] Colvin AC , West RV . Patellar instability. J Bone Jt Surg Am. 2008;90(12):2751–2762. 10.2106/JBJS.H.00211 19047722

[13] Cregar WM , Huddleston HP , Wong SE , Farr J , Yanke AB . Inconsistencies in reporting risk factors for medial patellofemoral ligament reconstruction failure: a systematic review. Am J Sports Med. 2022;50(3):867–877. 33914648 10.1177/03635465211003342

[14] Dan Milinkovic D , Schmidt S , Fluegel J , Gebhardt S , Zimmermann F , Balcarek P . Preoperative subjective assessment of disease‐specific quality of life significantly influenced the likelihood of achieving the minimal clinically important difference after surgical stabilization for recurrent lateral patellar instability. Knee Surg Sports Traumatol Arthrosc. 2025;33(1):86–95. 39031883 10.1002/ksa.12319 PMC11716333

[15] Desio SM , Burks RT , Bachus KN . Soft tissue restraints to lateral patellar translation in the human knee. Am J Sports Med. 1998;26(1):59–65. 9474403 10.1177/03635465980260012701

[16] Duchman KR , Bollier MJ . The role of medial patellofemoral ligament repair and imbrication. Am J Orthop (Belle Mead NJ). 2017;46(2):87–91. 28437493

[17] Elias JJ , Cosgarea AJ . Technical errors during medial patellofemoral ligament reconstruction could overload medial patellofemoral cartilage: a computational analysis. Am J Sports Med. 2006;34(9):1478–1485. 16685097 10.1177/0363546506287486

[18] Enderlein D , Nielsen T , Christiansen SE , Faunø P , Lind M . Clinical outcome after reconstruction of the medial patellofemoral ligament in patients with recurrent patella instability. Knee Surg Sports Traumatol Arthrosc. 2014;22(10):2458–2464. 25007722 10.1007/s00167-014-3164-5

[19] Feucht MJ , Mehl J , Forkel P , Achtnich A , Schmitt A , Izadpanah K , et al. Failure analysis in patients with patellar redislocation after primary isolated medial patellofemoral ligament reconstruction. Orthop J Sports Med. 2020;8(6):2325967120926178. 32613021 10.1177/2325967120926178 PMC7309400

[20] Galán‐Olleros M , Arviza‐Lorenzo P , Miranda‐Gorozarri C , Alonso‐Hernández J , Manzarbeitia‐Arroba P , Ramírez‐Barragán A , et al. Synthetic suture tape for medial patellofemoral ligament reconstruction is an effective treatment for complex paediatric patellofemoral instability. Knee Surg Sports Traumatol Arthrosc. 2024;32(11):2818–2829. 38746987 10.1002/ksa.12260

[21] Gobbi RG , Pereira CAM , Sadigursky D , Demange MK , Tírico LEP , Pécora JR , et al. Evaluation of the isometry of different points of the patella and femur for medial patellofemoral ligament reconstruction. Clin Biomech. 2016;38:8–12. 10.1016/j.clinbiomech.2016.08.002 27521477

[22] Gravesen KS , Kallemose T , Blønd L , Troelsen A , Barfod KW . Persistent morbidity after medial patellofemoral ligament reconstruction—a registry study with an eight‐year follow‐up on a nationwide cohort from 1996 to 2014. Knee. 2019;26(1):20–25. 30502935 10.1016/j.knee.2018.10.013

[23] Hardy A , Casabianca L , Andrieu K , Baverel L , Noailles T . Complications following harvesting of patellar tendon or hamstring tendon grafts for anterior cruciate ligament reconstruction: systematic review of literature. Orthop Traumatol Surg Res. 2017;103(8S):245–248. 28888527 10.1016/j.otsr.2017.09.002

[24] Hopper GP , Leach WJ , Rooney BP , Walker CR , Blyth MJ . Does degree of trochlear dysplasia and position of femoral tunnel influence outcome after medial patellofemoral ligament reconstruction? Am J Sports Med. 2014;42(3):716–722. 24458241 10.1177/0363546513518413

[25] Kang H , Zheng R , Dai Y , Lu J , Wang F . Single‐ and double‐bundle medial patellofemoral ligament reconstruction procedures result in similar recurrent dislocation rates and improvements in knee function: a systematic review. Knee Surg Sports Traumatol Arthrosc. 2019;27(3):827–836. 30136103 10.1007/s00167-018-5112-2

[26] Kernkamp WA , Wang C , Li C , Hu H , van Arkel ERA , Nelissen RGHH , et al. The medial patellofemoral ligament is a dynamic and anisometric structure: an in vivo study on length changes and isometry. Am J Sports Med. 2019;47(7):1645–1653. 31070936 10.1177/0363546519840278

[27] Kluczynski MA , Miranda L , Marzo JM . Prevalence and site of medial patellofemoral ligament injuries in patients with acute lateral patellar dislocations: a systematic review and meta‐analysis. Orthop J Sports Med. 2020;8(12):2325967120967338. 33403210 10.1177/2325967120967338 PMC7747126

[28] Krych AJ , O'Malley MP , Johnson NR , Mohan R , Hewett TE , Stuart MJ , et al. Functional testing and return to sport following stabilization surgery for recurrent lateral patellar instability in competitive athletes. Knee Surg Sports Traumatol Arthrosc. 2018;26(3):711–718. 28028569 10.1007/s00167-016-4409-2

[29] Le N , Blackman B , Zakharia A , Cohen D , de Sa D . MPFL repair after acute first‐time patellar dislocation results in lower redislocation rates and less knee pain compared to rehabilitation: a systematic review and meta‐analysis. Knee Surg Sports Traumatol Arthrosc. 2023;31(7):2772–2783. 36372845 10.1007/s00167-022-07222-w

[30] Lippacher S , Dreyhaupt J , Williams SRM , Reichel H , Nelitz M . Reconstruction of the medial patellofemoral ligament: clinical outcomes and return to sports. Am J Sports Med. 2014;42(7):1661–1668. 24758780 10.1177/0363546514529640

[31] Liu Z , Yi Q , He L , Yao C , Zhang L , Lu F , et al. Comparing nonoperative treatment, MPFL repair, and MPFL reconstruction for patients with patellar dislocation: a systematic review and network meta‐analysis. Orthop J Sport Med. 2021;9(9):23259671211026624. 10.1177/23259671211026624 PMC8485172 34604425

[32] Manjunath AK , Hurley ET , Jazrawi LM , Strauss EJ . Return to play after medial patellofemoral ligament reconstruction: a systematic review. Am J Sports Med. 2021;49(4):1094–1100. 32866030 10.1177/0363546520947044

[33] Matic GT , Magnussen RA , Kolovich GP , Flanigan DC . Return to activity after medial patellofemoral ligament repair or reconstruction. Arthroscopy. 2014;30(8):1018–1025. 24768468 10.1016/j.arthro.2014.02.044

[34] Mehl J , Otto A , Comer B , Kia C , Liska F , Obopilwe E , et al. Repair of the medial patellofemoral ligament with suture tape augmentation leads to similar primary contact pressures and joint kinematics like reconstruction with a tendon graft: a biomechanical comparison. Knee Surg Sports Traumatol Arthrosc. 2020;28(2):478–488. 31410528 10.1007/s00167-019-05668-z

[35] Melegari TM , Parks BG , Matthews LS . Patellofemoral contact area and pressure after medial patellofemoral ligament reconstruction. Am J Sports Med. 2008;36(4):747–752. 18296543 10.1177/0363546508314410

[36] Nomura E , Horiuchi Y , Inoue M . Correlation of MR imaging findings and open exploration of medial patellofemoral ligament injuries in acute patellar dislocations. Knee. 2002;9(2):139–143. 11950578 10.1016/s0968-0160(02)00002-9

[37] Panagiotopoulos E , Strzelczyk P , Herrmann M , Scuderi G . Cadaveric study on static medial patellar stabilizers: the dynamizing role of the vastus medialis obliquus on medial patellofemoral ligament. Knee Surg Sports Traumatol Arthrosc. 2006;14(1):7–12. 16001289 10.1007/s00167-005-0631-z

[38] Parikh SN , Nathan ST , Wall EJ , Eismann EA . Complications of medial patellofemoral ligament reconstruction in young patients. Am J Sports Med. 2013;41(5):1030–1038. 23539043 10.1177/0363546513482085

[39] Philippot R , Boyer B , Testa R , Farizon F , Moyen B . The role of the medial ligamentous structures on patellar tracking during knee flexion. Knee Surg Sports Traumatol Arthrosc. 2012;20(2):331–336. 21748394 10.1007/s00167-011-1598-6

[40] Puzzitiello RN , Waterman B , Agarwalla A , Zuke W , Cole BJ , Verma NN , et al. Primary medial patellofemoral ligament repair versus reconstruction: rates and risk factors for instability recurrence in a young, active patient population. Arthroscopy. 2019;35(10):2909–2915. 31604512 10.1016/j.arthro.2019.05.007

[41] Rosinski A , Chakrabarti M , Gwosdz J , McGahan PJ , Chen JL . Double‐bundle medial patellofemoral ligament reconstruction with allograft. Arthrosc Tech. 2019;8(5):e513–e520. 31194129 10.1016/j.eats.2019.01.011 PMC6552203

[42] Sallay PI , Poggi J , Speer KP , Garrett WE . Acute dislocation of the patella. A correlative pathoanatomic study. Am J Sports Med. 1996;24(1):52–60. 8638754 10.1177/036354659602400110

[43] Sappey‐Marinier E , Sonnery‐Cottet B , O'Loughlin P , Ouanezar H , Reina Fernandes L , Kouevidjin B , et al. Clinical outcomes and predictive factors for failure with isolated MPFL reconstruction for recurrent patellar instability: a series of 211 reconstructions with a minimum follow‐up of 3 years. Am J Sports Med. 2019;47(6):1323–1330. 31042437 10.1177/0363546519838405

[44] Schneider DK , Grawe B , Magnussen RA , Ceasar A , Parikh SN , Wall EJ , et al. Outcomes after isolated medial patellofemoral ligament reconstruction for the treatment of recurrent lateral patellar dislocations: a systematic review and meta‐analysis. Am J Sports Med. 2016;44(11):2993–3005. 26872895 10.1177/0363546515624673 PMC5502077

[45] Schöttle PB , Schmeling A , Rosenstiel N , Weiler A . Radiographic landmarks for femoral tunnel placement in medial patellofemoral ligament reconstruction. Am J Sports Med. 2007;35(5):801–804. 17267773 10.1177/0363546506296415

[46] Senavongse W , Amis AA . The effects of articular, retinacular, or muscular deficiencies on patellofemoral joint stability: a biomechanical study in vitro. J Bone Joint Surg Br. 2005;87–B(4):577–582. 10.1302/0301-620X.87B4.14768 15795215

[47] Servien E , Fritsch B , Lustig S , Demey G , Debarge R , Lapra C , et al. In vivo positioning analysis of medial patellofemoral ligament reconstruction. Am J Sports Med. 2011;39(1):134–139. 20929935 10.1177/0363546510381362

[48] Shah JN , Howard JS , Flanigan DC , Brophy RH , Carey JL , Lattermann C . A systematic review of complications and failures associated with medial patellofemoral ligament reconstruction for recurrent patellar dislocation. Am J Sports Med. 2012;40(8):1916–1923. 22679297 10.1177/0363546512442330 PMC3615712

[49] Smirk C , Morris H . The anatomy and reconstruction of the medial patellofemoral ligament. Knee. 2003;10(3):221–227. 12893143 10.1016/s0968-0160(03)00038-3

[50] Smith TO , Walker J , Russell N . Outcomes of medial patellofemoral ligament reconstruction for patellar instability: a systematic review. Knee Surg Sports Traumatol Arthrosc. 2007;15(11):1301–1314. 17684729 10.1007/s00167-007-0390-0

[51] Steensen RN , Dopirak RM , McDonald WG . The anatomy and isometry of the medial patellofemoral ligament: implications for reconstruction. Am J Sports Med. 2004;32(6):1509–1513. 15310579 10.1177/0363546503261505

[52] Stephen JM , Kittl C , Williams A , Zaffagnini S , Marcheggiani Muccioli GM , Fink C , et al. Effect of medial patellofemoral ligament reconstruction method on patellofemoral contact pressures and kinematics. Am J Sports Med. 2016;44(5):1186–1194. 26944575 10.1177/0363546516631736

[53] Stephen JM , Lumpaopong P , Deehan DJ , Kader D , Amis AA . The medial patellofemoral ligament: location of femoral attachment and length change patterns resulting from anatomic and nonanatomic attachments. Am J Sports Med. 2012;40(8):1871–1879. 22729504 10.1177/0363546512449998

[54] Tanaka MJ , Chahla J , Farr J , LaPrade RF , Arendt EA , Sanchis‐Alfonso V , et al. Recognition of evolving medial patellofemoral anatomy provides insight for reconstruction. Knee Surg Sports Traumatol Arthrosc. 2019;27(8):2537–2550. 30370440 10.1007/s00167-018-5266-y

[55] Thaunat M , Erasmus PJ . Recurrent patellar dislocation after medial patellofemoral ligament reconstruction. Knee Surg Sports Traumatol Arthrosc. 2008;16(1):40–43. 17973099 10.1007/s00167-007-0418-5

[56] Tompkins MA , Arendt EA . Patellar instability factors in isolated medial patellofemoral ligament reconstructions—what does the literature tell us? A systematic review. Am J Sports Med. 2015;43(9):2318–2327. 25748469 10.1177/0363546515571544

[57] Webster KE , Wittwer JE , O'Brien J , Feller JA . Gait patterns after anterior cruciate ligament reconstruction are related to graft type. Am J Sports Med. 2005;33(2):247–254. 15701611 10.1177/0363546504266483

[58] Yoo YS , Chang HG , Seo YJ , Byun JC , Lee GK , Im H , et al. Changes in the length of the medial patellofemoral ligament: an in vivo analysis using 3‐dimensional computed tomography. Am J Sports Med. 2012;40(9):2142–2148. 22837430 10.1177/0363546512453301

[59] Ziegler CG , Fulkerson JP , Edgar C . Radiographic reference points are inaccurate with and without a true lateral radiograph: the importance of anatomy in medial patellofemoral ligament reconstruction. Am J Sports Med. 2016;44(1):133–142. 26561652 10.1177/0363546515611652

[60] Zimmermann F , Privalov M , Franke J , Grützner PA , Balcarek P , Vetter SY (2023) Reconstruction of the medial patellofemoral ligament with nonresorbable suture tape normalizes patellar maltracking independent of patella‐side fixation technique. Knee Surg Sports Traumatol Arthrosc 31(7):2870–2876 36454291 10.1007/s00167-022-07256-0

